# A survey of invasive plants on grassland soil microbial communities and ecosystem services

**DOI:** 10.1038/s41597-020-0422-x

**Published:** 2020-03-09

**Authors:** Jennifer K. Bell, Steven D. Siciliano, Eric G. Lamb

**Affiliations:** 1 Soil Science Department, University of Saskatchewan, Saskatoon, SK S7N 5A8 Canada; 2 Plant Sciences Department, University of Saskatchewan, Saskatoon, SK S7N 5A8 Canada

**Keywords:** Microbial ecology, Grassland ecology, Invasive species, Soil microbiology, DNA sequencing

## Abstract

Invasive plants can cause changes in the structure and function of the ecosystem being invaded. Any changes in ecosystem diversity and community composition will likely alter ecosystem services provided by that ecosystem. However, how these ecosystem services may change is poorly understood. To elucidate how these ecosystem services will change with invasion, we sampled 561 plots undergoing invasion by smooth brome (*Bromus inermis*) and four other invasive species at a native Rough Fescue prairie located near Saskatoon, Saskatchewan, Canada. Soil and plant surveys were undertaken weekly for 26 weeks between May of 2014 and November of 2014, or the growing season. We measured a suite of ecosystem services, including greenhouse gasses, extracellular enzyme function, forage production, glyphosate degradation and decomposition. Furthermore, soil physical and chemical properties were measured, and soil bacterial and fungal communities were sequenced. This is a large and multifaceted dataset with complex temporal and spatial attributes which can be used to answer numerous questions regarding the functioning of prairie ecosystems and how invasive species will impact that functioning.

## Abstract (summary)

**Table:** 

Measurement(s)	invasive species • native species • ecosystem decay • greenhouse gas • DNA
Technology Type(s)	Survey • monitoring device • DNA sequencing
Factor Type(s)	geographic location • sampling date
Sample Characteristic - Environment	prairie • soil
Sample Characteristic - Location	Saskatoon

Machine-accessible metadata file describing the reported data: 10.6084/m9.figshare.11898213

## Background & Summary

Invasive species can have severe impacts on the ecosystem being invaded, including altering the plant community with subsequent cascading effects on ecosystem services. Changes in plant species composition affect the quantity and quality of root exudates to the soil^1–3^ alter rates of nutrient cycling^4–6^, and change inputs of organic matter to the soil^1^. Despite an increasing knowledge of how invasive plants change these soil properties, relatively little is known about how invasive plants influence soil microbial community structure and function^2,4,5,7,8^. Changes in the chemical and physical properties of soil, as well as in litter and exudate inputs, are almost certain to impact microbial community assembly processes and community composition, which in turn, will alter ecosystem services.

Smooth brome invasion into intact native grasslands has been extensively studied^9–17^. Smooth brome invasion radically changes plant community structure^15,16^, and subsequently causes changes in soil nutrient cycling and soil microbial community structure and function^9–11,18,19^. Changes in nutrient cycling and microbial community structure and function will have cascading and possibly long-term effects on the ecosystem services provided by native prairies. Furthermore, any changes caused by smooth brome invasion will likely help to perpetuate the invasion, impeding efforts to restore native prairie plant populations.

The complexity of brome-soil interactions opens many research avenues. Our initial work on this topic was based on a small (60 plot) dataset collected at a single time point in mid-growing season. This simple dataset has led to six separate publications^9–11,18–20^. These previous studies inspired the dataset presented here, where we measure the impacts of smooth brome and four other invasive plant species on soil communities and processes in a 561 plot data set with sampling spanning the entire growing season, and including the measurement of a wide range of ecosystem services. For each of the 561 plots measured, bacterial and fungal communities were sequenced, and greenhouse gas emissions, extracellular enzymes, glyphosate degradation and soil physical and chemical properties were measured. Additionally, plant surveys and biomass data were collected at each plot. This dataset is not only large, but also offers a comprehensive study of prairie soil processes in an ecosystem undergoing invasion. We anticipate this dataset will be of interest to ecologists in disciplines beyond our own (plant ecology and soil microbiology) and wish to make it available for those studies.

## Methods

### Site description

Kernen Prairie is a 130 ha remnant rough fescue prairie situated on the edge of the city of Saskatoon, Saskatchewan, Canada (5°10″N, 106° 33″W). The site supports grassland and low shrub communities, and includes a small number of aspen (*Populus tremuloides*) bluffs and ephemeral wetlands (Coupland and Brayshaw 1953, Pylypec 1986). Common native grass species include Plains Rough Fescue (*Festuca hallii*), Wheatgrass (*Elymus lanceolatus*), and Needlegrass (*Hesperostipa curtiseta*). Common native broadleaf species include Northern Bedstraw (*Galium boreale*), Pasture Sage (*Artemisia frigida)*, and Prairie Rose (*Rosa arkansana)*. Low shrub communities dominated by Western Snowberry *(Symphoricarpos occidentalis)* are also common. The prairie is undergoing multiple invasions by species including the forage grasses Smooth Brome (*Bromus inermis*) and Kentucky Bluegrass (*Poa pratensis*), and the forbs Canada Thistle (*Cirsium arvense*), perennial sow thistle (*Sonchus arvensis*), and Absinthe (*Artemisia absinthum*) (Slopek and Lamb 2017). Microtopography, soil water availability, prescribed fire, and grazing history are primary influences on the plant community structure of the prairie^21–25^.

### Study design

**Fig. 1:**
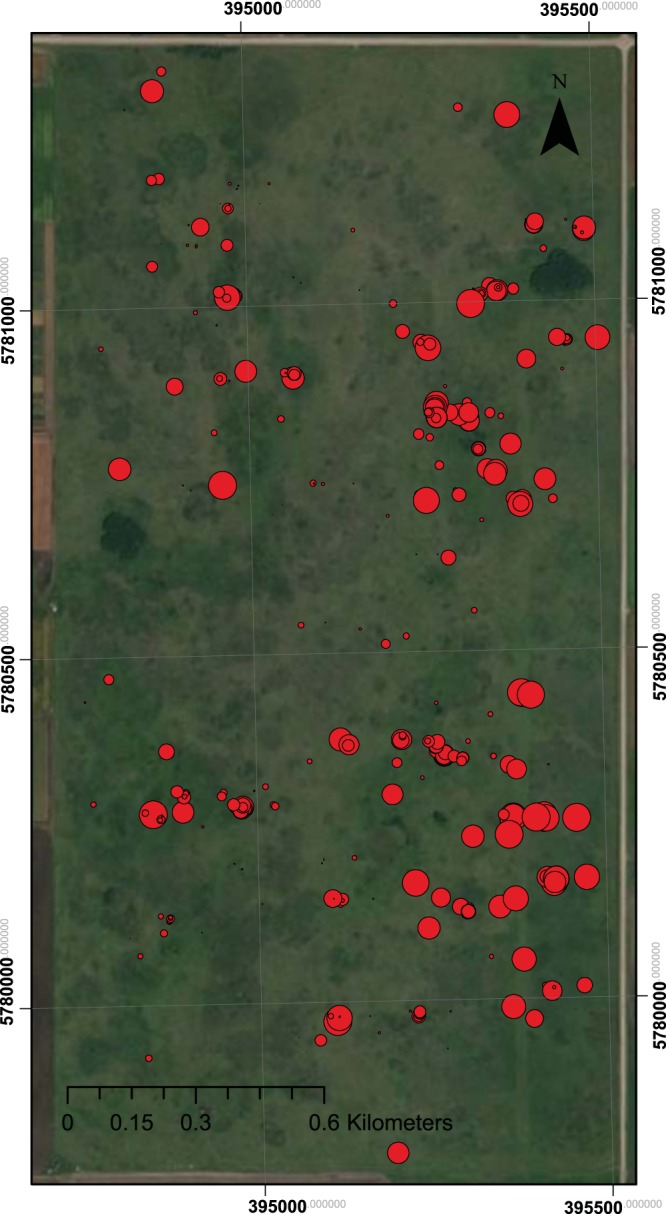
Kernen Prairie with sites sampled scaled for intensity of invasion based on percent of cover that was categorized as invasive out of the total plant biomass present at that sampling point. Axes are eastings (30000s) and northings (50000).

Samples were located in clusters designed to capture the major spatial patterns at the site using modified Fibonacci spirals (Fig. 1)^26^. This is a spatially explicit sampling design that ensures sufficient samples are taken across a range of lag distances, enabling univariate and multivariate spatial analyses including spatially explicit structural equation modeling (SE-SEM)^27^. In this study the lag distance ranges included the scales of plant - soil relationships (0–25 cm), plant community effects (1–2 m), edaphic and large herbivore effects (10–20 m), topography and plant dispersal (50–100 m), and landform and land use history (250–500 m), capturing ecological mechanisms from the root zone of a single plant to entire the landscape^20,28–31^.

Each modified Fibonacci spiral used in this study consisted of 13 sampling points (Fig. 1). The raw spiral included ~40,000 potential points at intervals of 10 cm. The 13 points selected were designed to capture the spatial scales listed above. Two clusters of sample points near the center of the spiral (one cluster of 3 points in a line spaced 10 cm apart at the center of the spiral and a single point 1 m away, the other, 2 points 14 cm apart and a third at a distance of 1 m) near the center of the spiral served to capture the scales of plant-soil and plant-community relationships. Further points at distances of up to 250 m from the spiral center served to capture lag distances out to 500 m within a single spiral.

Two Fibonacci spirals were sampled weekly from May 15, 2014 to November 5, 2014. One spiral each week was centered on a smooth brome patch, and the other was centered in grassland that had not been invaded by brome. Native spirals were located on randomly selected points. Random points were constrained to ensure that the most distant plots within the spiral were within the boundary of the prairie. Brome invaded points were identified on an initial survey of Kernen Prairie in early spring 2014 where the prairie was walked on an ~50 m grid, and all smooth brome patches spotted were marked located using a GPS. As brome frequency, and hence the density of brome patches marked with GPS, varied across the prairie, a stratified random approach was used to select smooth brome points to be spiral centers (Fig. 1). Specifically, the prairie was divided equally into nine 260 m by 530 m zones. A zone was randomly selected, and then a random brome point was selected for sampling from that zone. All zones were sampled before a second point was selected from any particular zone.

**Fig. 2:**
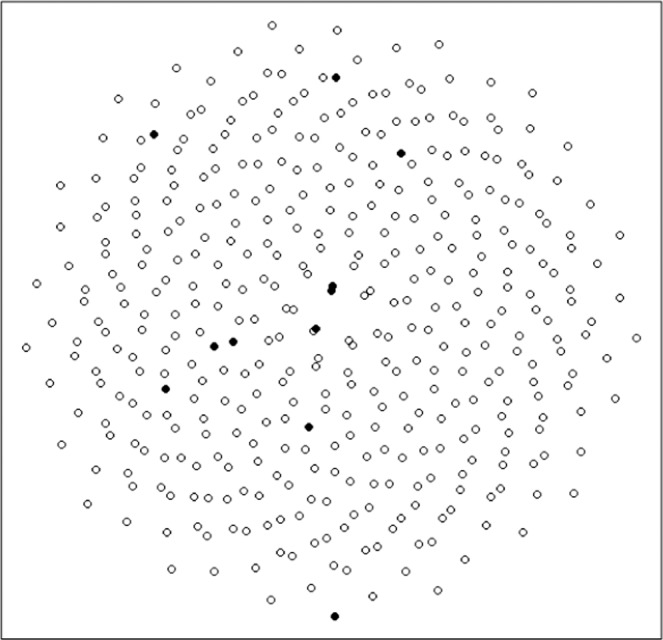
Sampling design of 10 points selected from a small Fibonacci spiral. The closest points are 20 cm apart; the furthest 25 m apart. Increasing the number of points captures distances up to 300 m. Scaling up interpoint distances to kms can locate sites for small spirals.

The sampling design above resulted in a total of 26 weekly sampling plots. All 26 weekly sampling plots were collected, however adverse weather conditions and sampling crew size limited the number that could be sampled in some weeks. When constraints presented, sample plots distant from the center were omitted first. In cases where a sample plot fell in a wetland or in the middle of an Aspen (*Populus tremuloides*) bluff, the plot was not sampled (Fig. 2).

### Soil sampling

Soil temperature and moisture was measured in the field using a 5TE Decagon probe (Pullman, Washington). A 1 cm diameter, 15 cm depth core of soil was taken with a push corer in each plot for future DNA work and frozen at −80 °C until processing. Two cores were collected (5 cm diameter, 30 cm depth) with a slide hammer corer, and soil was passed through a 4.5 mm sieve. Sieved soil was then collected for soil aggregate analysis and nutrient analysis. Soil was stored at 4 °C until processing.

### Plant surveys

Plant species and litter were collected from a 50 by 50 cm quadrat centered on each plot. In the case of closely spaced points, the plots overlapped. In such cases, subplots separating the overlapping and non-overlapping parts of plots were clipped. In each plot and subplot, each vascular plant species was clipped separately, dried at 60 °C and weighed. Dry plant biomass was summed into native, invasive, and total biomass and by functional group to get a plot measures of plant biomass^32^. Litter (dead plant material from the previous growing season) was also collected and weighed. Plant biomass was adjusted for live versus senesced biomass beginning on August 15^th^. When clipping, percent greenness of the biomass was estimated for each species. Plant community evenness was calculated using the E_var_ index^33^. Forage quality (poor, fair, good) was assigned for each species using established descriptions^34,35^.

Sampling points were initially classified as native or invaded based on the origin point of the Fibonacci spiral; however individual plots were later reclassified as native or invaded based on the proportion of invaded biomass present^32^. Plots with greater than 25% invaded biomass were classified as invaded giving 274 native plots and 287 invaded plots (Fig. 2). The dataset was then further subdivided into three seasons based on plant biomass with green-up encompassing weeks 1–8 (May 15–July 3), peak biomass from weeks 9–18 (July 14–September 10) and senescence from weeks 19–26 (September 24–November 5).

### Greenhouse gas sampling

Probes attached to a DX-4015 Fourier transform infrared trace gas analyzer (FTIR-TGA, Gasmet Technologies Oy, Helsinki, Finland), were used to concurrently measure subsurface soil concentrations of carbon dioxide (CO_2_), methane (CH_4_) and nitrous oxide (N_2_O)^32^. The FTIR was linked to an opaque LI-COR chamber using a 10 m by 3 mm Teflon sample and control lines. The chamber used had an enclosed area of 0.0314 m^2^ and was attached to a 20 cm collar that had been inserted into the soil to a depth of approximately 7 cm. Gas fluxes were measured for ten minutes with one-minute sample intervals^36^.

### Glyphosate degradation

Glyphosate degradation was measured using 5 grams of field fresh soil to which 0.01 µM of ^13^C-labelled glyphosate was placed into 160 ml serum bottles. At 0, 1, 3, and 5 days, a syringe was used to sample gas in the headspace and inject it into two sets of vacutainers. One set was analyzed with a Scion 456-GC for total CO_2_, while the second set was analyzed for the ratio of ^13^CO_2_ to ^12^CO_2_ with a Picarro G2201-I analyzer. The concentration of ^13^CO_2_ from the degradation of glyphosate was calculated in nmol per g soil per day^32^.

### Soil physical and chemical properties

Soil moisture content was measured using a Mettler Toledo Moisture Analyzer MJ33 (Colombus, Ohio) (Table 1). Five grams of sieved, field fresh soil was added for each plot sampled. Soil pH was measured by adding 10 ml 0.01 M CaCl_2_ to 5 g air-dried soil. The soil slurry then sat at room temperature for 30 minutes and was stirred four to five times during the incubation period. After this period, the slurry was left to settle for 30 minutes and then measured using a pH meter^32^.

**Table 1:** A summary of the mean and standard deviation in parenthesis of soil physical and chemical properties by treatment and season.

Treatment	Season	pH	% Soil Moisture	250% μM aggregate	53% μM aggregate	% Clay/Silt	Inorganic Phosphorous (mg/kg)	Water Extractable Carbon (mg/kg)	Water Extractable Nitrogen (mg/kg*)*
Native	Green-up	6.86(0.47)	26.18(6.61)	54.1(13.3)	26.2(8.6)	19.6(11.4)	6.95(6.89)	345.34(166.2)	161.29(107.6)
	Peak	6.84(0.44)	23.38(5.36)	50.2(16.2)	33.6(9.0)	16.2(8.4)	5.40(2.38)	206.02(132.8)	109.01(132.9)
	Senescence	7.01(0.39)	17.57(4.14)	57.2(13.9)	31.5(9.9)	11.3(4.9)	8.33(10.83)	205.61(120.2)	195.59(147.3)
Invaded	Green-up	6.88(0.37)	27.34(4.63)	55.6(14.0)	24.8(7.8)	19.5(10.5)	7.09(2.64)	321.60(172.3)	177.60(116.3)
	Peak	6.88(0.42)	20.89(5.75)	57.5(16.1)	31.2(10.7)	11.4(6.8)	5.89(3.18)	233.16(131.8)	115.83(139.9)
	Senescence	7.10(0.49)	17.59(4.79)	61.6(13.5)	29.0(9.6)	9.4(4.7)	7.89(9.65)	248.45(127.6)	212.43(125.1)

Total organic carbon and nitrogen as well as inorganic nitrogen was extracted by shaking 10 grams of field fresh soil with 50 ml of 2 M KCl for 1 hour at 20 °C on a rotary shaker at 200 rpm. The extracts were then centrifuged at 2000 rpm for ten minutes at room temperature. Following centrifugation, the extracts were filtered through Whatman Grade #2 110 mm filter paper and frozen at −20 °C until analysis. The extracts were then run on a Shimadzu TOC-VCPH with a TNM-L attachment (Shimadzu Corporation, Kyoto, Japan)^32^.

Inorganic and total phosphorous were measured using a modified sodium bicarbonate protocol^37^. Two grams of air-dried soil was added to an acid washed flask with 50 ml 0.5 M NaHCO_3_ and was shaken for room temperature at 120 rpm for sixteen hours. Samples were then centrifuged at room temperature for 20 minutes at 5000 rpm and filtered through a 0.45 µm filter. For total phosphorous, 5 ml of the filter solution was added an acid washed glass test tube following 0.5 g ammonium persulfate and 10 ml 0.9 M H_2_SO_4_. The test tubes were then autoclaved and frozen until further analysis. For inorganic phosphorous, 10 ml of filtered sample was added along with 6 ml of 0.9 M H_2_SO_4_ to acidify to pH 1.5. Samples were then incubated at 4 °C for half an hour and filtered through Whatman Grade #2 110 mm filter paper and frozen at −20 °C until analysis. The amount of total and inorganic phosphate was determined colorimetrically using the ammonium molybdate-antimony potassium tartrate-ascorbic acid method^37^. Organic phosphorus was calculated as the difference between total extractable phosphorous and extractable inorganic phosphorus^32^.

Wet aggregate stability was measured using a method adapted from Soil Sampling and Methods of Analysis^37^ using an oscillating dual-layered sieve machine modified from Six *et al.*^38^. The samples were kept at field moisture and gently sieved to 2 mm with roots and plant material removed. A 250 μm sieve was stacked on a 53 μm sieve and then were submersed in deionized water. Soil (5 g) was added to the top of each sieve set and allowed settle for 2 minutes. After 2 minutes, the sieves were oscillated 50 times (18.75 oscillations min^−1^; 2.58 cm s^−1^). Floating debris was removed with vacuum suction and subtracted from the total soil added. The remaining water and soils from the three sets of sieves were composited and then dried in the oven at 60 °C overnight, weighed and placed into one of three size groups: >250 μm, 250–53 μm and <53 μm sized aggregates^32^.

### Soil extracellular enzymes and decomposition

Soil phosphatase and arylsulfatase analyzed following well established methods^39,40^. Field fresh soil (0.1 g) was weighed and 20 µL of toluene was added. Samples were then incubated at room temperature for 1 hour. After incubation, 400 µL of 0.5 M acetate buffer at pH 5.8 was added. For phosphatase, 100 µL of 10 mM p-nitrophenyl phosphate was added. For arylsulfatase, 100 µL of 10 mM p-nitrophenyl was added. Tubes were mixed and incubated at 37 °C for 1 hour. After incubation, the tubes were placed on ice to stop the reaction for 5 minutes and then centrifuged at 14000 RPM for 2 minutes. 150 µL of the supernatant was added to a 96-well plate that contained 100 µL of 0.5 M NaOH solution in each well. The wells were mixed and read at 405 nm using an iMark microplate reader (Bio Rad, Hercules, California)^32^.

Soil dehydrogenase was analyzed following Trevors^41^. Field fresh soil (0.1 g) was weighed and 40 µL of water and 20 µL of 0.4% Iodonitrotetrazolium chloride solution was added. Samples were then incubated in the dark for 48 hours at room temperature. After incubation 1 ml of methanol was added, mixed for 30 seconds and centrifuged at 14000 RPM for 2 minutes. After centrifugation, 250 µl of the supernatant was placed in a 96-well plate and read at 480 nm using an iMark microplate reader (Bio Rad, Hercules, California)^32^.

Potential nitrification was measured using a test media which was prepared with 4 mM (NH_4_)_2_SO_4_ as a growth substrate, 15 mM NaClO_3_ as an oxidation inhibitor, and 1 mM KHPO_4_ as a buffer^37^. Field fresh soil was incubated for a week at 20 °C and then air dried for 24 hours at room temperature. After drying 5 g of soil was added to a 125 ml serum bottle and 125 ml of the test media was added. The serum bottles were shaken at 125 rpm a 10 °C for 36 hours. Samples were removed from the serum bottle at 12, 24, and 36 hours by removing 2 ml of the soil/test media slurry. The sample was then added to a 15 ml falcon tube containing 2 ml 4 M KCl to terminate the reaction and centrifuged for 5 minutes at 5000 rpm. The supernatant was then filtered through a 0.45 μm syringe tip filter. Nitrite production was measured using a colorimetric method. The color reagent was prepared using 100 ml 85% phosphoric acid, 10 g sulfanilamide and 1 g n-(1-naphthyl)-ethylenediamine dihydrochloride dissolved into 1000 ml deionized water. Nitrite standards were prepared using liquid nitrogen and were run at a 0.2–2 mM gradient. To trigger color development, 10 µl of the color reagent was added to 240 µl of sample or standard and mixed in a 96 well plate. The plate was then read at 543 nm^32^.

Microfaunal feeding rates were measured using bait lamina sticks^42^. The sticks were created using styrene strips, 120 mm in length, six mm wide and one mm thick. Sixteen holes with 1.5 mm diameter were added 5 mm apart from each other, beginning 10 mm from the base of the stick. The bait was prepared using cellulose, bran flakes and active carbon at a 70:27:3 ratio by weight with enough tap water added to make a paste. The paste was then pressed into the holes on each stick and left to dry overnight. Sticks were placed in each plot in the top layer of soil and removed after two weeks. Sticks were scanned and the average number of holes consumed were counted over the three-week period^32,43^.

### Root exudates

Root exudates were quantified by taking one gram of soil from the push corer, which was then weighed into an Erlenmeyer flask and 10 ml 0.05 M NaCl was added. The slurry was then shaken at 180 rpm for 15 minutes. The slurry was decanted into a 15 ml falcon tube and centrifuged at 5000 rpm for 15 minutes. Samples were then stored at −20 °C until further analysis. Once ready to analyze, the samples were filtered using Whatman Grade #2 filter to remove any remaining soil or roots. After filtering samples were adjusted to a pH between 6 and 7 using 0.5 M HCl or NaOH when appropriate. For the solid phase extraction, strata X-AW 33 µm Polymeric Weak Anion cartridges (Phenomenex, Torrance, California) were prepared by being placed on a vacuum manifold and rinsed with 6 ml each of methanol and then Milli-Q water. After preparation, the sample was added to the cartridge followed by 10 ml of 25 mM ammonium acetate. The vacuum was applied until the resin was dry and the samples were stored at −20 °C until further analysis. Following the ammonium acetate, 4 mL was added to the cartridge and allowed to soak through for two minutes followed by 5 minutes of vacuum. New 15 ml falcon tubes were placed under then cartridge and two 2.5 ml aliquots of 5% ammonium hydroxide in methanol was added. The resin was allowed to dry between aliquots. The tubes were then placed in a RapidVap vacuum dry evaporation system (Labconco, Kansas City, Missouri) at 40 °C and allowed to dry completely. The dry samples were then resuspended in 5 ml 10 mM KOH, vortexed and transferred to vials for analysis on the Ion Chromatograph (IC).

Root exudate samples were run on an ion chromatograph (DIONEX IC2000, Thermo Fisher Scientific, Waltham, MA) and a Dionex AS-DV auto-sampler (Thermo Fisher Scientific, Waltham, MA). A Dionex IonPac AS18 column was used (Thermo Fisher Scientific, Waltham, MA), 4 by 250 mm and the effluent source was Dionex effluent generator cartage (EGC) III potassium hydroxide (KOH) (Thermo Fisher Scientific, Waltham, MA). The cell temperature was 35 °C and column temperature was 30 °C. Other conditions included a 1 mL/ min flow rate, 25 µl injection volume, a 3 loop overfill factor, a push full inject mode, an ASRS 3004 mm suppressor, and a 34-minute run time. A multistep gradient was used starting at 10 mM and increasing to 50 mM over 30 minutes. Quality control was insured by running malate, succinate, tartrate, oxalate and citrate standards separately and together ranging from 0 to 25 ppm. A set of standards was run at the beginning, end, and every 10 samples in addition to a blank. Samples were frozen in a −20 °C freezer prior to IC analysis^32,44^.

### DNA extraction and amplification

The plant root and soil microbial communities were measured from a 5 g subsample of soil with roots from the pencil core. Samples were ground at 22.5 hertz for five minutes on a Retsch Mixer Mill MM 440 (Germany). Between samples, chambers were shaken with bleach for five minutes to minimize the carryover of DNA.

DNA was extracted from 250 mg rhizosphere soil (soil in direct contact with the roots) using Qiagen PowerSoil extraction kit (Hilden, Germany) following manufacturer instructions. Extraction duplicates were included. All wells were spiked with a known concentration of *Aliivibrio fischeri* as an internal standard^45^. After extraction, DNA was tested for quantity following the standard Qubit protocol (Thermo Fisher Scientific, Waltham Massachusetts). Prior to amplification, DNA was standardized to 1 ng/μl.

Bacterial diversity was assessed by amplifying the V4 region of the bacterial 16S rRNA using the primer set 515 F with Illumina adapters (5′TCGTCGGCAGCGTCAGATGTGTATAAGAGACAGGTGYCAGCMGCCGCGGTAA3′) and the 806R (5′-GTCTCGTGGGCTCGGAGATGTGTATAAGAGACAG GGA CTA CCG GGG TAT CT-3′)^46^. The PCR reaction mix consisted of 12.5 μl Invitrogen Platinum SuperFi PCR master mix (Thermo Fisher Scientific, Waltham, Massachusetts), 0.1 μl of each primer and 10.3 μl nuclease free water, and 2 μl of the standardized template DNA. The PCR conditions were 95 °C for 5 minutes as an initial denaturation, followed by 95 °C for 30 seconds, 54 °C for 30 seconds, 72 °C for 30 seconds for 35 cycles, and a final elongation of 72 °C for 7 minutes. Negative controls and PCR duplicates were included.

Fungal diversity was assessed by amplifying using the primer set ITS1F (5′-CTTGGTCATTTAGAGGAAGTAA–3′) and ITS2 (5′-GCTGCGTTCTTCATCGATGC–3′)^47^. The PCR reaction mix consisted of 12.5 μl Invitrogen Platinum Green Hot Start PCR master mix (Thermo Fisher Scientific, Waltham, Massachusetts) 1 μl of each primer and 8.5 μl nuclease free water, and 2 μl of the standardized template DNA. The PCR conditions were the same as the bacterial change with the expectation of a change in annealing temperature of 51 °C, rather than 54 °C. Fungi was amplified using a different taq polymerase because attempts to amplify bacteria using Platinum Green were unsuccessful. Finally, an annealing gradient was performed for both fungi to optimize amplification and the lack of non-specific amplification was verified using an agarose gel and base pair ladder.

The plant root community was assessed by amplifying the trnL operon (trnL-F: 5′-CGAAATYGGTAGACGCTACG-3′ and trnL-R: 5′-CCDTYGAGTCTCTGCACCTATC-3′)0^16^. The PCR reaction mix consisted of 12.5 μl Invitrogen Platinum SuperFi PCR master mix (Thermo Fisher Scientific, Waltham, Massachusetts), 1 μl of each primer, 8.5 μl nuclease free water, and 2 μl of the standardized template DNA. The PCR conditions were identical to the bacterial PCR except for a change in annealing temperature from 54 °C to 62 °C.

In all cases, PCR product was purified to eliminate primers and impurities using 1:1 ratio of Nucleomag NGS clean-up and size select (D-mark Biosciences, Scarborough, Ontario). After purification, samples were indexed following the Illumina protocol^48^, purified again to remove excess index primers, quantified and standardized to 4 nM, and pooled. Pooled libraries were then sequenced using the Illumina MiSeq platform.

For bacteria, a total of 8,506,841 reads were produced with an average of 12,008 reads per sample^49^. Sequences were imported into QIIME2 v 2019.1^50^ and primers were removed using *cutadapt*^51^. Reads were then processed into amplicon sequence variants (ASVs) using DADA2^52^ resulting in 263,940 ASVs with an average of 13,055 ASVs per sample. ASVs were then classified using a 515/806 trained Greengenes database^53^ classifier. For fungal sequences, a total of 12,371,309 reads were generated with an average of 21,478 reads per sample^49^. Fungal primers were also imported into QIIME2, primers were removed using *cutadapt* and sequences were sorted into ASVs using DADA2 resulting in a total of 17,374 ASVs with an average of 4,735 ASVs per sample. Fungal ASVs were then classified using the Unite Database^54^.

qPCR was performed to get an estimate of bacterial abundances using QuantStudio 7 Flex (Applied Biosystems, California, USA). The reaction mix consisted of 11 μl SYBR Green (Qiagen, Hilden, Germany), 1 μl each of 515 F and 806 R primers that were also used for sequencing, 5 μl water and 2 μl DNA template. The Touchdown qPCR conditions were a hold stage consisting of 50 °C for two minutes, followed by 95 °C for ten minutes. The touchdown stage was 11 cycles of 95 °C for 1 minute, a beginning temperature of 65 °C for one minute, increasing by 1 °C for each cycle, and a final temperature of 72 °C for one minute. The PCR stage was 40 cycles and consisted of 85 °C for one-minute stage, followed by a data acquisition step of 55 °C for one minute and a temperature of 72 °C for one minute. The melt curve stage was 95 °C for one minute, 60 °C for one minute and a final temperature of 95 °C with data acquisition occurring during the ramp. A known quantity of *A. fischeri* was used to create the standard curve using copy numbers. qPCR was not performed for fungi for several reasons. First, fungi can be unicellular or multicellular making it difficult to get an accurate number of organisms. Additionally, ITS primers have large biases^55^ meaning that any qPCR to estimate fungal community abundance would be inaccurate.

## Potential Use

This dataset is large and can be used in a variety of ways to examine prairie ecosystem functioning. For example, the greenhouse gas emission data could be used separately to examine emissions over the course of a growing season as affected by exotic plant invasion. The enzyme data coupled with the soil physical and chemical data also has the potential to be used separately to study how nutrient cycling takes place throughout a prairie when the ground is not frozen and how nutrient cycling is impacted by invasion. The microbial datasets are large and can be used to answer a large range of questions from how to microbial communities assemble, both with or without invasion, to how microbial communities are linked to differences in soil enzymatic profiles and gas emissions. Finally, this whole dataset can be used to provide a comprehensive look at how changing plant communities impact ecosystem functioning as whole, from spring thaw to winter freezing. The dataset presented is so rich in soil, plant and microbial data that any question that one would like to ask about prairie plant and soil functioning can be examined.

## Data Records

All raw sequence files can be found at the National Center for Biotechnology Information (NCBI) under Bioproject PRJNA580515^49^. Compiled soil physical and chemical properties, along with plant survey data and other ecosystem services can be found in the Dryad repository, along with the ASVs estimates, which have been normalized using the *A. fischeri* internal standard (10.5061/dryad.1ns1rn8q7)^32^. Sequence files are named with the week collected first, followed by the plot. For example, the label 1–901 indicates week one, plot 901. Plant measurements with _g in the name have been adjusted for greenness. Standing biomass includes dead plant biomass.

## Technical Validation

All DNA samples were verified using the qubit standard protocol and PCR products were verified using an agarose gel. Sequence library concentrations were verified using the standard qubit protocol.
